# Evaluation of Serum Neudesin Levels in Patients With Adrenal Incidentaloma

**DOI:** 10.7759/cureus.85968

**Published:** 2025-06-13

**Authors:** Faruk Recep Özalp, Ayfer Çolak, Anıl Baysoy, Selin Onur, Veli İyilikçi, Hamiyet Yılmaz Yaşar

**Affiliations:** 1 Medical Oncology, Dokuz Eylül University, Izmir, TUR; 2 Biochemistry, İzmir Tepecik Training and Research Hospital, Izmir, TUR; 3 Biochemistry, Izmir City Hospital, Izmir, TUR; 4 Biochemistry, Başakşehir Çam ve Sakura City Hospital, Istanbul, TUR; 5 Biochemistry, Gaziantep City Hospital, Gaziantep, TUR; 6 Endocrinology, Diabetes and Metabolism, Izmir City Hospital, Izmir, TUR

**Keywords:** adrenal incidentaloma, adrenal incidentaloma investigations, clinical biomarker, hormonal activity, neudesin

## Abstract

Background: Neudesin is a neurotrophic factor involved in various metabolic and inflammatory pathways. However, its role in adrenal incidentalomas remains unclear. This study aimed to evaluate serum neudesin levels in patients with adrenal incidentalomas and investigate their potential clinical significance.

Methodology: This is a single-center, cross-sectional, controlled study conducted in the Endocrinology Clinic of Izmir Tepecik Training and Research Hospital between July 2018 and August 2019. The study included 68 patients diagnosed with adrenal incidentaloma and a control group of 38 healthy individuals. Age, gender, body mass index (BMI), adrenocorticotropic hormone (ACTH), cortisol, dehydroepiandrosterone sulfate (DHEA-S), total testosterone, plasma aldosterone, plasma renin activity, 24-hour urine metanephrine and normetanephrine levels, and neudesin levels were measured, and comorbidities were recorded.

Results: The patient group was significantly older and had a higher BMI than the control group (*P *< 0.001). Neudesin levels were considerably higher in the patient group (5.7 ± 1.5 ng/mL) compared to the control group (4.26 ± 1.92 ng/mL) (*P* < 0.001). Neudesin levels correlated with age, BMI, and the presence of incidentalomas. Multiple regression analysis revealed that neudesin correlated with only the presence of adrenal incidentaloma (Beta = 1.12, *P* = 0.005).

Conclusions: Our findings suggest that serum neudesin levels may be associated with adrenal incidentalomas and related metabolic alterations. Further studies should investigate the potential diagnostic or prognostic value of neudesin in adrenal disorders.

## Introduction

Adrenal incidentalomas are masses larger than 10 mm that are discovered incidentally in the adrenal glands during imaging studies performed for unrelated reasons [1]. With the increased use of imaging modalities such as computed tomography (CT) and magnetic resonance imaging (MRI), the detection rate of adrenal incidentalomas has increased [2]. The prevalence of adrenal incidentalomas on imaging studies is estimated to be around 4% in the general population and increases with age. While many of these lesions are benign, a subset may have hormonal activity or malignant potential and require further evaluation [3].

Patients with adrenal incidentalomas should be assessed to determine whether the lesion is hormonally active or malignant [4]. Hormonal activity commonly presents as mild autonomous cortisol secretion, pheochromocytoma, or primary aldosteronism [5]. The detection of hormone activity often requires advanced diagnostic testing, including biochemical assays and dynamic endocrine testing, making the clinical evaluation process more complex and time-consuming [6]. In addition, differentiating benign from malignant adrenal masses is critical, as adrenocortical carcinoma is a rare but aggressive malignancy with a poor prognosis [7].

Neudesin is a secreted protein that belongs to the membrane-associated progesterone receptor (MAPR) family [8]. It consists of 172 amino acids and is encoded by a gene located on chromosome 1p33 [9]. This protein is predominantly expressed in the central nervous system, particularly in the hypothalamus, where it contributes to the regulation of appetite, energy balance, and metabolic homeostasis [10]. In addition, neudesin expression has been associated with several cancers, including glioma, breast and lung cancer, suggesting a potential role in tumorigenesis [11]. Experimental studies have shown that neudesin modulates neuronal survival through the activation of protein kinase A (PKA) and phosphoinositide 3-kinase (PI3K) signaling pathways. Previous studies have reported that the neurotrophic activity of neudesin is dependent on the binding of heme to the cytochrome b5-like heme/steroid binding domain [8]. Previous research has indicated that neudesin expression is influenced by hormonal changes, particularly those related to steroidogenesis [12]. Given the role of cytochrome b5 in adrenal steroidogenesis, we hypothesized that there might be a relationship between hormonal activity and neudesin levels in adrenal incidentalomas [13]. This raises the possibility that neudesin levels could serve as a biomarker for adrenal tumors, helping to differentiate between hormonally active and inactive adrenal incidentalomas. However, data on its role in adrenal pathophysiology remain limited.

In this study, we aimed to compare neudesin levels between a healthy control group and patients with adrenal adenomas and investigate the relationship with hormonal and metabolic parameters. By investigating this relationship, we aim to gain insight into whether neudesin can serve as a biomarker in the evaluation of adrenal incidentalomas.

## Materials and methods

This was a single-center, cross-sectional, controlled study conducted at the Endocrinology Clinic of Izmir Tepecik Training and Research Hospital between July 2018 and August 2019.

Inclusion criteria included age over 18 years, presence of an adrenal incidentaloma, absence of active malignancy, no diagnosis of neuropathy, and willingness to participate in the study. Exclusion criteria were age under 18 years, pregnancy, a BMI less than 18 or greater than 30, presence of active malignancy, presence of prediabetes or diabetes, and unwillingness to participate. Population size was calculated using G Power 3.1 by evaluating the mean neudesin level in previous studies (alpha: 0.05, power: 95%) [14]. It was determined that 28 patients were required for both groups. The study included 38 controls and 68 patients. After analysis, 5 of 68 patients were found to be hormonally active. Of the five hormone-active patients, one had Cushing's syndrome, two had mild autonomous cortisol hypersecretion, and two had pheochromocytoma. The age, sex, body mass index (BMI), blood pressure, and comorbidities (hypertension) of the patients were recorded. BMI was calculated using the formula: weight (kg)/height² (m). Routine laboratory tests included cortisol levels after a 1 mg dexamethasone suppression test, dehydroepiandrosterone sulfate (DHEA-S), total testosterone, plasma aldosterone, plasma renin activity, and 24-hour urinary metanephrine and neudesin levels. Blood samples for neudesin were collected at 10 am and stored at -20 °C. Measurements were performed using a Beckman® Coulter Unicel DXI 800 autoanalyzer (Beckman Coulter Inc., Brea, CA), a Shimadzu® high-performance liquid chromatography system (Shimadzu Corporation, Kyoto, Japan), and a Siemens® Immulite XPI autoanalyzer (Siemens Healthcare Diagnostics Inc., Tarrytown, NY). Neudesin levels were determined using the Cloud-Clone® Corp. Human ELISA Kit (Cloud-Clone Corp., Wuhan, China). The intra-assay and inter-assay coefficients of variation were 5.4 % and 6.1 % for neudesin (ng/mL), respectively. CT and MRI were used for incidentaloma diagnosis and localization. This study was approved by the Ethics Committee of Izmir Tepecik Training and Research Hospital (Decision number: 2018/8-17).

Statistical analysis

Continuous variables were expressed as the mean ± standard deviation, while categorical variables were presented as percentages. The normality of the data was assessed using the Shapiro-Wilk test. Chi-square and Fisher's exact tests were used for categorical variables, while the Mann-Whitney U test was applied for continuous variables. Correlation analysis and multiple linear regression were performed to examine relationships between continuous variables. The statistical analyses were conducted using the SPSS Statistics 25.0 for iOS software program (IBM Corp., Armonk, NY) and R software (The R Foundation for Statistical Computing, Vienna, Austria). Statistical significance was set at *P *< 0.05.

## Results

There was no statistically significant difference between the two groups in gender distribution (*P* = 0.535). However, considerable variations were observed in age and BMI values. Patients were found to be significantly older, with an average age of 52.13 ± 10.92 years compared to 34.89 ± 13.27 years in the control group (*P* < 0.001). In addition, the patient group exhibited a higher BMI (27.30 ± 2.58 kg/m²) in comparison to the control group (23.89 ± 3.49 kg/m²). This discrepancy was found to be statistically significant (*P* < 0.001). The proportions of right, left, and bilateral localization were 42.6%, 44.1%, and 13.2%, respectively (Table 1).

**Table 1:** Patient characteristics. *P* < 0.05. ^a^Chi-square test. ^b^Chi-square value.

Parameter	Control group (*n* = 38, %)	Patient group (*n* = 68, %)	*P*-value	Test statistics
Age (years)			<0.001^a^	44.7^b^
18-39	29 (76.3)	8 (11.8)		
40-64	7 (18.4)	49 (72.1)		
≥65	2 (5.3)	11 (16.2)		
Gender			0.535^a^	0.481^b^
Female	22 (57.9)	44 (64.7)		
Male	16 (42.1)	24 (35.3)		
Body mass index (BMI) (kg/m^2^)			<0.001^a^	24.9^b^
<25	25 (65.8)	12 (17.6)		
25-30	13 (34.2)	56 (82.4)		
Localization				
Right	-	29 (42.6)		
Left	-	30 (44.1)		
Bilateral	-	9 (13.2)		
Total	38 (100)	68 (100)		

Plasma aldosterone and renin activity were measured exclusively in the patient group, with levels recorded at 6.98 ± 1.88 ng/dL and 6.88 ± 2.91 ng/mL/hour, respectively. With regard to laboratory parameters (Table 2), ACTH showed no significant differences between groups (*P* = 0.107). Similarly, cortisol and urinary metanephrine levels were not significantly different (*P* = 0.748, *P* = 0.169, *P* = 0.07). However, the patient group had significantly lower DHEA-S levels (102.54 ± 70.63 mcg/dL) in comparison to the control group (210.24 ± 149.38 mcg/dL) (*P* < 0.001). While total testosterone levels were marginally lower in the patient group (140.45 ± 144.41 ng/dL) than in the control group (188.26 ± 192.89 ng/dL), the difference was not statistically significant (*P* = 0.163). Conversely, neudesin levels exhibited a marked increase in patients (5.70 ± 1.50 ng/mL) in comparison to controls (4.26 ± 1.92 ng/mL) (*P* < 0.001).

**Table 2:** Table 2. Comparison of mean age, BMI, and laboratory parameters between patient and control groups. ^a^Mann-Whitney U value. *P < 0.05. ACTH, adrenocorticotropic hormone; BMI, body mass index; DHEA-S, dehydroepiandrosterone sulfate

Parameter (Mean ± SD)	Control group	Patient group	*P*-value*	Test statistics
Age (years)	34.89 ± 13.27	52.13 ± 10.92	<0.001*	2171.5^a^
BMI (kg/m²)	23.89 ± 3.49	27.30 ± 2.58	<0.001*	2044^a^
ACTH (pg/mL)	13.08 ± 10.87 (*n *= 11)	20.73 ± 11.75	0.107	107^a^
Cortisol (mcg/dL)	1.18 ± 0.36	1.43 ± 2.33	0.748	1243.5^a^
DHEA-S (mcg/dL)	210.24 ± 149.38	102.54 ± 70.63	<0.001*	659^a^
Total testosterone (ng/dL)	188.26 ± 192.89	140.45 ± 144.41	0.163	1064^a^
Plasma aldosterone (ng/dL)	-	6.98 ± 1.88 (*n *= 6)	-	
Plasma renin activity (ng/mL/hour)	-	6.88 ± 2.91 (*n *= 6)	-	
24-hour urinary metanephrine (µg/24 hours)	133.29 ± 79.55	180.44 ± 240.08	0.169	1501^a^
24-hour urinary normetanephrine (µg/24 hours)	264.37 ± 161.95	399.72 ± 516.70	0.07	1567.5^a^
Neudesin (ng/mL)	4.26 ± 1.92	5.70 ± 1.50	<0.001*	1905.5^a^

Correlation analyses were performed between neudesin and all other parameters. Neudesin correlated with age (*r* = 0.322, *P* < 0.001), BMI (*r* = 0.406, *P* = 0.017), DHEA-S (*r* = -0.232, *P* = 0.017), and the presence of incidentalomas (*r* = 0.406, *P* < 0.001). No correlation was found between neudesin levels and other parameters. Multiple regression analysis showed that only the presence of an incidentaloma significantly contributed to neudesin levels (Beta = 1.12, *P* = 0.005) (Table 3).

**Table 3:** Multiple regression analysis evaluating the effects of age, BMI, DHEA-S levels, and presence of incidentaloma on neudesin levels (R² = 0.540). *P-value of < 0.05 was considered as significant. β, unstandardized regression coefficient; CI, confidence interval; BMI, body mass index; DHEA-S, dehydroepiandrosterone sulfate

Parameter	β	95%CI (Min-Max)	*P*-value
Age	0.002	-0.083 to 0.084	0.995
BMI	-0.485	-0.633 to 0.046	0.085
DHEA-S	0.305	-0.003 to 0.013	0.241
Presence of incidentaloma	1.12	1.57 to 7.58	0.005*

In addition, the Tukey HSD test showed that neudesin levels were significantly higher in the incidentaloma group compared to the control group (*P* < 0.001). However, there was no significant difference in neudesin levels between the control group and the hormonally active incidentaloma group (*n* = 5; *P* = 0.735), nor between the incidentaloma group and the hormonally active incidentaloma group (*P* = 0.461). In conclusion, incidentalomas had higher neudesin levels than controls, but hormonally active incidentalomas were not significantly different from the control group.

## Discussion

This is the first study investigating the relationship between neudesin levels and adrenal incidentalomas. The potential of neudesin as a biomarker for screening hormonally active cases was evaluated. Our results showed that neudesin levels were increased in adrenal incidentalomas. Due to the insufficient number of patients (*n* = 5) in the hormonally active group, the relationship between hormone activity and neudesin could not be established.

In our study, we found that neudesin was positively correlated with BMI, but this was not significant in the regression analysis. This may be due to the small sample size or the confounding effect of other variables in the regression model. Vergani et al. found that neudesin levels in obese children were similar to our findings [15]. Çelikkol et al. and Kratochvilova et al. found that neudesin levels were inversely correlated with obesity [16,17]. As there are conflicting data in the literature on the relationship between BMI and neudesin levels, further studies are needed. Similar to BMI, we found a positive correlation between neudesin and age, but this was not significant in regression analysis. The studies conducted by Yılmaz Yaşar et al. and Karataş et al. revealed no correlation between age and neudesin level [18,19]. This discrepancy may be attributable to the abnormal age distribution observed between the control and patient groups or the limited number of patients included in the study. 

In the present study, neudesin levels were shown to be significantly elevated in the patient group compared to the control group (*P* < 0.001). In the course of the analysis, the hormonally active patients in the patient group were considered as a separate group. This analysis showed that neudesin levels were equivalent in the hormonally active patients and the control group. This discrepancy may be due to the limited sample size of the hormonally active group. An alternative explanation might be the inclusion of pheochromocytoma cases within the hormonally active group, while the remaining patients had mild autonomous cortisol secretion. Given the association of neudesin with steroidogenesis, the presence of patients diagnosed with pheochromocytoma in the hormonally active group may have a negative effect on the results of the analysis.

In two separate studies of patients diagnosed with polycystic ovary syndrome, a correlation was found between progesterone levels and neudesin levels [18,20]. Given that progesterone production is in the initial stages of steroidogenesis, an increase in neudesin may be an expected result in hormonally active adrenal incidentalomas [21].

The PKA pathway plays an important role in the development of adrenal adenomas [22-24]. Considering that neudesin activates the PKA and PI3K pathways, the high level of neudesin in the adrenal adenoma group can be explained [10].

This study has several limitations. First, the relatively small sample size may affect the generalizability of the findings. Second, although patients with diabetes mellitus, prediabetes, or active malignancy were excluded, detailed information on other comorbidities (e.g., hypertension, metabolic syndrome) and medication use (e.g., antihypertensives, statins, corticosteroids) was not systematically collected. These factors may have influenced neudesin levels and led to residual confounding. Finally, due to its cross-sectional design, this study cannot establish a causal relationship between neudesin levels and adrenal incidentalomas; longitudinal studies are required to determine whether neudesin plays a direct role in tumor development or progression.

## Conclusions

These findings highlight the potential role of neudesin as a biomarker in adrenal incidentalomas, though the underlying mechanisms remain unclear. Future studies with larger cohorts and mechanistic investigations are needed to determine whether neudesin plays a causative role in adrenal incidentalomas development or is merely an associated biomarker. Understanding this relationship could provide new insights into the metabolic and endocrine implications of adrenal tumors.

## References

[1] Jing Y, Hu J, Luo R (2022). Prevalence and characteristics of adrenal tumors in an unselected screening population: a cross-sectional study. Ann Intern Med.

[2] Anagnostis P, Karagiannis A, Tziomalos K, Kakafika AI, Athyros VG, Mikhailidis DP (2009). Adrenal incidentaloma: a diagnostic challenge. Hormones (Athens).

[3] Young WF Jr (2007). Clinical practice. The incidentally discovered adrenal mass. N Engl J Med.

[4] Grumbach MM, Biller BM, Braunstein GD (2003). Management of the clinically inapparent adrenal mass ("incidentaloma"). Ann Intern Med.

[5] Fassnacht M, Tsagarakis S, Terzolo M (2023). European Society of Endocrinology clinical practice guidelines on the management of adrenal incidentalomas, in collaboration with the European Network for the Study of Adrenal Tumors. Eur J Endocrinol.

[6] Lee JM, Mee KK, Seung-Hyun K (2017). Guidelines for the management of adrenal incidentaloma: The Korean Endocrine Society, Committee of Clinical Practice Guidelines. Korean J Med.

[7] Kasperlik-Załuska AA, Otto M, Cichocki A (2008). Incidentally discovered adrenal tumors: a lesson from observation of 1,444 patients. Horm Metab Res.

[8] Kimura I, Nakayama Y, Yamauchi H (2008). Neurotrophic activity of neudesin, a novel extracellular heme-binding protein, is dependent on the binding of heme to its cytochrome b5-like heme/steroid-binding domain. J Biol Chem.

[9] Kimura I, Yoshioka M, Konishi M, Miyake A, Itoh N (2005). Neudesin, a novel secreted protein with a unique primary structure and neurotrophic activity. J Neurosci Res.

[10] Ohta H, Kimura I, Konishi M, Itoh N (2015). Neudesin as a unique secreted protein with multi-functional roles in neural functions, energy metabolism, and tumorigenesis. Front Mol Biosci.

[11] Kimura I, Nakayama Y, Zhao Y, Konishi M, Itoh N (2013). Neurotrophic effects of neudesin in the central nervous system. Front Neurosci.

[12] Kimura I, Nakayama Y, Konishi M, Terasawa K, Ohta M, Itoh N, Fujimoto M (2012). Functions of MAPR (membrane-associated progesterone receptor) family members as heme/steroid-binding proteins. Curr Protein Pept Sci.

[13] Storbeck KH, Swart AC, Goosen P, Swart P (2013). Cytochrome b5: novel roles in steroidogenesis. Mol Cell Endocrinol.

[14] Polkowska A, Pasierowska IE, Pasławska M, Pawluczuk E, Bossowski A (2019). Assessment of serum concentrations of adropin, afamin, and neudesin in children with type 1 diabetes. Biomed Res Int.

[15] Vergani E, Bruno C, Cipolla C, Currò D, Mancini A (2022). Plasma levels of neudesin and glucose metabolism in obese and overweight children. Front Endocrinol (Lausanne).

[16] Çelikkol A, Binay Ç, Ayçiçek Ö, Güzel S (2022). Serum neudesin levels in obese adolescents. J Clin Res Pediatr Endocrinol.

[17] Kratochvilova H, Lacinova Z, Klouckova J (2019). Neudesin in obesity and type 2 diabetes mellitus: the effect of acute fasting and weight reducing interventions. Diabetes Metab Syndr Obes.

[18] Yilmaz Yasar H, Demirpence M, Colak A, Zeytinli M, Yasar E, Taylan A (2022). Serum neudesin levels in patients with polycystic ovary syndrome. Ginekol Pol.

[19] Karatas O, Calan M, Yuksel A (2023). The level of the neudesin in type-2 diabetic patients and the relationship between the metabolic parameters and carotid intima-media thickness. Minerva Endocrinol (Torino).

[20] Bozkaya G, Fenercioglu O, Demir İ, Guler A, Aslanipour B, Calan M (2020). Neudesin: a neuropeptide hormone decreased in subjects with polycystic ovary syndrome. Gynecol Endocrinol.

[21] Kater CE, Giorgi RB, Costa-Barbosa FA (2022). Classic and current concepts in adrenal steroidogenesis: a reappraisal. Arch Endocrinol Metab.

[22] Kamilaris CD, Stratakis CA (2018). An update on adrenal endocrinology: significant discoveries in the last 10 years and where the field is heading in the next decade. Hormones (Athens).

[23] Sherlock M, Scarsbrook A, Abbas A, Fraser S, Limumpornpetch P, Dineen R, Stewart PM (2020). Adrenal Incidentaloma. Endocr Rev.

[24] Wu L, Xie J, Qi Y (2022). Mutational landscape of non-functional adrenocortical adenomas. Endocr Relat Cancer.

